# Involvement of human chorionic gonadotropin in regulating vasculogenic mimicry and hypoxia-inducible factor-1α expression in ovarian cancer cells

**DOI:** 10.1186/s12935-016-0327-0

**Published:** 2016-06-24

**Authors:** Min Su, Xiangxiang Xu, Weiwei Wei, Sainan Gao, Xiaoying Wang, Caoyi Chen, Yuquan Zhang

**Affiliations:** Department of Obstetrics and Gynecology, The Affiliated Hospital of Nantong University, No 20, Xisi Rd, Nantong, 226001 People’s Republic of China; Suzhou Municipal Hospital, Suzhou, China; Changzhou 2nd People’s Hospital, Changzhou, China; The Immunology Laboratory of Nantong University, Nantong, China; Department of Genetics, College of Life Sciences, Nantong University, Nantong, Jiangsu China

**Keywords:** Ovarian cancer, Human chorionic gonadotropin, Vasculogenic mimicry, Hypoxia inducible factor-1α

## Abstract

**Background:**

Human chorionic gonadotropin (hCG) can play a crucial role in angiogenesis. In the present study, we focused on hCG to gain insight into its potential effects on vasculogenic mimicry (VM) in ovarian cancer cells.

**Methods:**

Ovarian cancer OVCAR-3 cells were incubated with different concentrations of recombinant hCG in 3-dimensional cultures. VM was identified by morphological observations and vascular endothelial cell marker detection in OVCAR-3 cells. Expression of hCG, hypoxia-inducible factor-1α (HIF-1α), and the endothelial cell markers CD31, VEGF, and factor VIII were detected by reverse transcription polymerase chain reaction and western blotting. The effect of hCG on endothelial cell-marker expression in ovarian cancer cells was further explored using small interfering RNA (siRNA) and plasmid-based approaches.

**Results:**

Incubation of OVCAR-3 cells with recombinant hCG induced vessel-like network formation, which was accompanied by significant elevation of vascular marker expression. Attenuation of hCG expression by siRNA in OVCAR-3 cells suppressed the expression of endothelial cell markers and HIF-1α by tumour cells. Overexpression of hCG in OVCAR-3 cells resulted in increased expression of endothelial cell markers and HIF-1α.

**Conclusions:**

HCG was crucial for changing the phenotype of OVCAR-3 cells to endothelial-like cells. The effect of hCG induction on VM in ovarian cancer cells is potentially associated with HIF-1α.

## Background

The concept of vasculogenic mimicry (VM) was introduced in 1999 and was described as the unique ability of highly aggressive melanoma cells to obtain endothelial-like characteristics and form de novo vascular-like networks. The aggressive tumor cells have the potential to express vascular marker in this novel microcirculation [1]. Tumour cells have direct access to the bloodstream through the tumour cell-lined vessels and tend to spread aggressively due to VM formation [2]. The presence of VM correlates with an increased risk for metastasis and, therefore, poor clinical outcomes [3]. VM has been reported in ovarian cancer, breast cancer, prostate cancer, myeloma, hepatocellular carcinoma, Ewing’s sarcoma, and renal clear cell carcinoma [3–9]. The underlying pathogenic mechanisms of VM are unclear, but the influence of the tumour microenvironment is potentially associated with VM formation. Hypoxia was reported to promote VM formation in 3-dimensional (3D) cultures through the hypoxia inducible factor-1α (HIF-1α) pathway [10, 11].

Choriocarcinoma, which is noted to have high-level human chorionic gonadotropin (hCG) production, is also characterized by the presence of a multitude of haemorrhagic channels, similar to VM. Recently, we reported that ovarian cancer cells can express endothelium-associated genes to form vasculogenic-like networks in 3D gels in a microenvironment containing added hCG [12, 13]. HCG belongs to a family of glycoprotein hormones characterized by a heterodimeric structure with an α-subunit non-covalently bound to the β-subunit, the latter being hormone specific [14]. Although β-hCG is normally expressed at detectable levels during pregnancy, it is also ectopically synthesized in trophoblastic and non-trophoblastic carcinomas of the colon, prostate, bladder, breast, lung, and ovaries [15, 16]. β-hCG has recently been proposed as a biomarker of poor prognosis in cancer [17–19]. It has been suggested that placental hCG and vascular endothelial growth factor (VEGF) interact during formation of the placental vasculature [20]. Ectopically produced hCG has recently been found to exhibit angiogenic growth factor properties that are central to cancer progression [16]. β-HCG expression in cervical cancer is associated with the extent of tumour vascularisation [21]. Serum hCG levels have recently been linked to neo-vascularisation of non-seminomatous testicular germ cell tumours [22]. However, little has been reported regarding the effects of hCG on VM.

We hypothesised that hCG may play a crucial role in the development of VM in ovarian cancer. In this study, we explored the possible effects of hCG on VM in the hCG receptor-positive ovarian cancer cell line OVCAR-3 in a 3D angiogenesis system. OVCAR-3 cells were incubated with different concentrations of hCG to evaluate the influence of hCG on VM formation. HCG receptor-negative ovarian cancer SKOV3 cells were used as a control. We identified VM by morphological observations and detected vascular marker expression. A small interfering RNA (siRNA) against hCG mRNA and a phCMV1-derived hCG expression vector were used to gain insight into the potential effects of hCG on transendothelial differentiation and HIF-1α expression in OVCAR-3 cells.

## Results

### Vascular cell marker expression and morphological flexibility induced by hCG in OVCAR-3 cells

**Fig. 1:**
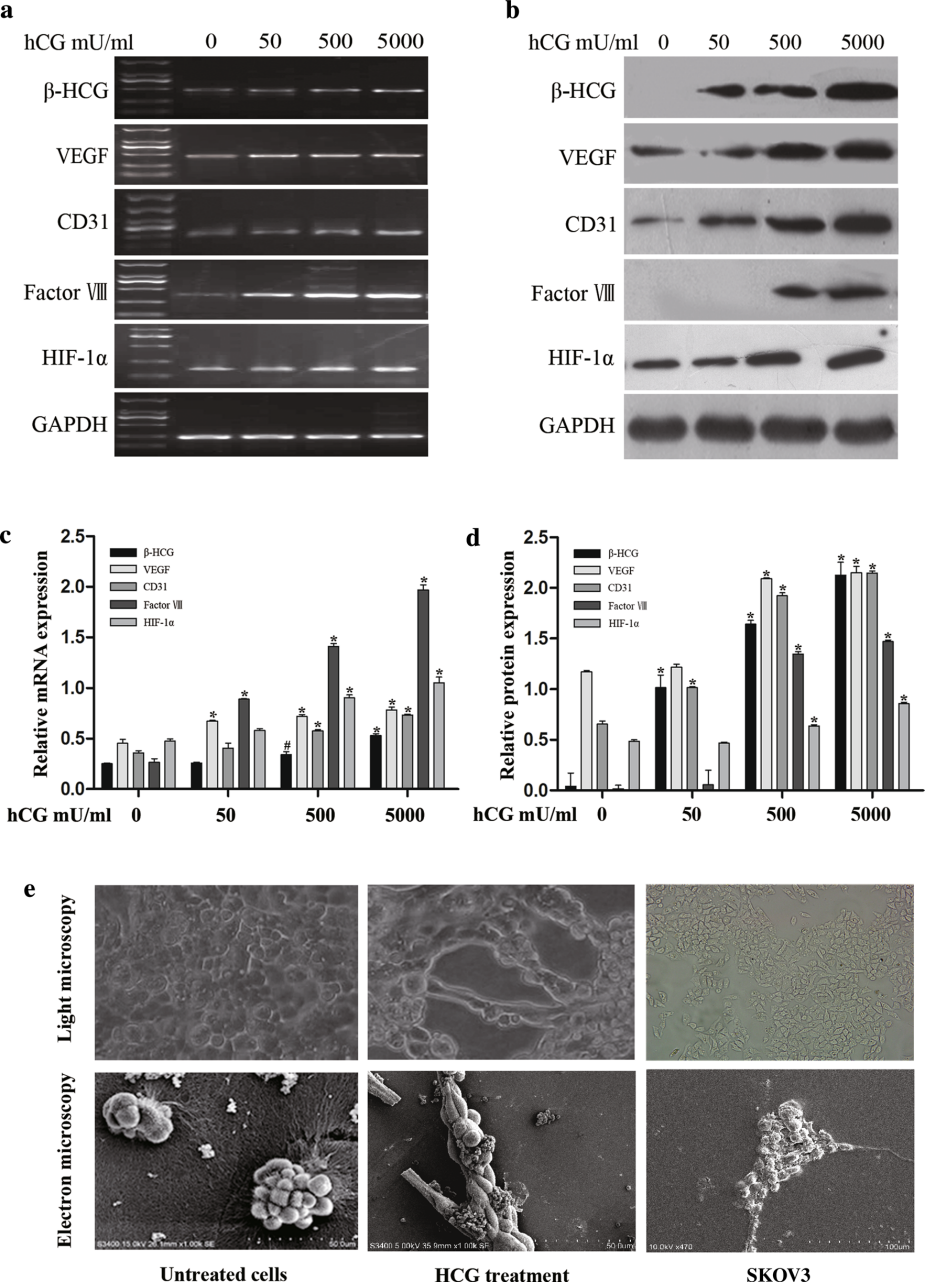
Expression of vascular cell markers and hCG in and morphological flexibility of hCG-treated OVCAR-3 cells. **a**, **b** Expression levels of vascular markers CD31, VEGF, factor VIII, hCG, and HIF-1α were determined in OVCAR-3 cells exposed to 50, 500, or 5000 mU/ml hCG for 7 days. HCG treatment stimulated the expression of vascular markers and HIF-1α in OVCAR-3 cells in a dose-dependent manner. **a** The mRNA levels were analysed by RT-PCR. **b** Protein levels were detected by western blotting. **c**, **d** Band densities were quantified by densitometric analysis. Protein and mRNA content was quantified for 3 independent replicates and the data are presented as the mean ± SD. The data shown are presented after normalization with GAPDH expression and were analysed using 1-way ANOVA. *p < 0.01, ^#^p < 0.05. **e** Light and scanning-electron microscopy observations showed tubular network and channel formation by OVCAR-3 cells in the 3D matrix after exposure to 5000 mU/ml hCG. Representative morphological changes are shown

OVCAR-3 cells were incubated in 3D gels with increasing concentrations of hCG (50, 500, or 5000 mU/ml) for 7 days. The expression of vascular cell markers in OVCAR-3 cells was analysed by reverse transcription-polymerase chain reaction (RT-PCR) and western blotting. As shown in Fig. 1a, the expression levels of CD31, VEGF, factor VIII mRNA and HIF-1α increased significantly in response to hCG treatment, in a dose-dependent manner, as did their respective protein-expression levels (Fig. 1b). The highest dose of hCG (5000 mU/ml) showed the most significant effect. We also found that the relative expression of hCG in OVCAR-3 cells significantly increased in response to hCG treatment in a dose-dependent manner, compared with that observed in unstimulated cells (Fig. 1a–d). However, hCG treatment did not significantly increase expression of the vascular cell marker in SKOV-3 cells.

OVCAR-3 cells displayed considerable plasticity in cell shape when embedded in the 3D matrix under hCG treatment when observed by light and scanning-electron microscopy. Tubular network and channel formation with OVCAR-3 cells were observed in the 3D gel exposed to 5000 mU/ml hCG (Fig. 1e). The effects in the 3D gel on exposure to 50 or 500 mU/ml hCG with respect to morphological changes were not obvious, compared with the appearance of untreated cells. SKOV-3 cells failed to form tubular networks or channels in the 3D gel, even when exposed to 5000 mU/ml hCG.

### Inhibition of vascular marker and HIF-1α expression in OVCAR-3 cells by β-hCG siRNA

**Fig. 2:**
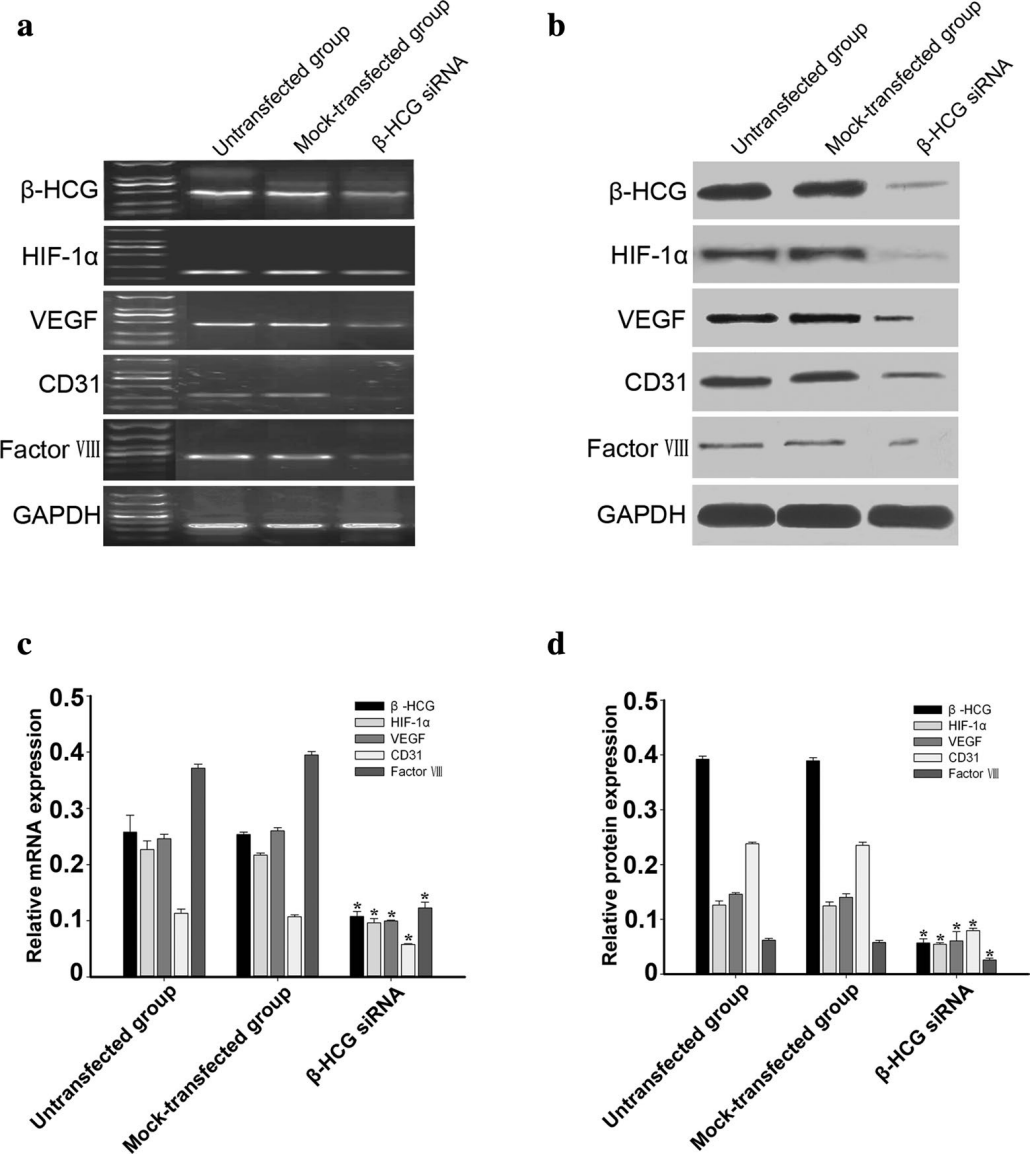
Inhibition of hCG expression using siRNA resulted in suppressed vascular marker and HIF-1α expression. **a**, **b** Expression of hCG in OVCAR-3 cells was inhibited by siRNA targeting hCG mRNA, but not by a negative control siRNA. Compared with untransfected OVCAR-3 cells and mock-transfected OVCAR-3 cells, the expression of CD31, VEGF, factor VIII, and HIF-1α decreased in OVCAR-3 cells transfected with hCG siRNA. **a** mRNA expression of the vascular cell marker, HIF-1α and hCG was analysed by RT-PCR. **b** Protein expression of the vascular cell marker, HIF-1α and hCG was analysed by western blotting. **c**, **d** Band densities were quantified by densitometric analysis. Protein and mRNA content measured in 3 independent replicates was quantified and the data are presented as the mean ± SD. The data shown are presented after normalization with GAPDH bands and analysed by 1-way ANOVA. *p < 0.01

The specificity of the effect of hCG was further assessed by down-regulating β-hCG expression with siRNA. β-hCG siRNA specifically suppressed hCG expression. HCG mRNA expression decreased by 71.87 % and hCG protein expression decreased by 85.39 %. Our data showed that expression of vascular cell markers in OVCAR-3 cells was inhibited effectively by β-hCG siRNA. For example, expression of CD31, VEGF, factor VIII mRNA decreased by 57.36, 77.05, and 86.2 %, respectively, in OVCAR-3 cells transfected with β-hCG siRNA, compared with the negative control group. β-hCG siRNA also reduced CD31, VEGF, Factor VIII protein expression by 82.68, 71.05, and 69.05 %, respectively. HIF-1α mRNA and protein expression was also decreased by 69.53 and 70.61 %, respectively (Fig. 2; p < 0.01).

### Expression of vascular markers and HIF-1α in OVCAR-3 cells with up-regulated β-hCG expression

**Fig. 3:**
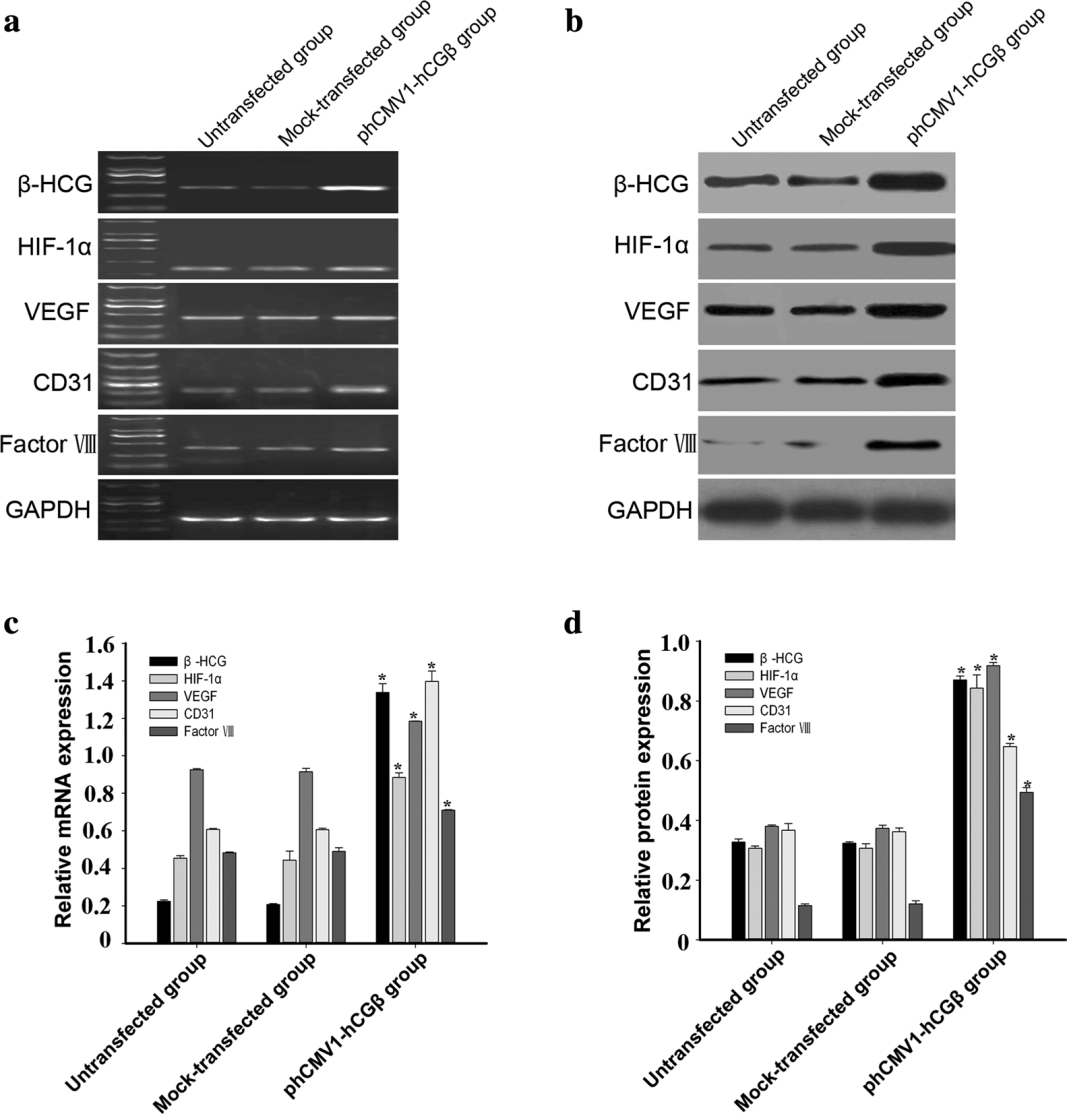
Up-regulated expression of vascular markers and HIF-1α in OVCAR-3 cells transfected with the phCMV1 vector expressing β-hCG (phCMV1-hCGβ). **a**, **b** HCG expression increased significantly in transfected OVCAR-3 cells, as determined by RT-PCR and western blot analysis. Compared with untransfected and mock-transfected cells, both mRNA (**a**) and protein expression (**b**) of CD31, VEGF, Factor VIII, and HIF-1α increased significantly. **c**, **d** Band densities were quantified by densitometric analysis. Protein and mRNA content measured in 3 independent replicates was quantified and the data are presented as the mean ± SD. The data shown were normalized to GAPDH bands and analysed by 1-way ANOVA. *p < 0.01

To further investigate the effect of hCG on the expression of vascular markers in ovarian cancer cells, OVCAR-3 cells were transfected with the phCMV1 vector expressing β-hCG. HCG mRNA and protein expression in transfected OVCAR-3 cells was verified by RT-PCR and western blot analysis. Compared with parental and vector control cells, a higher level of hCG expression was detected in transfectants overexpressing hCG. HCG expression increased by 6.5-fold at the mRNA level and 2.7-fold at the protein level. The angiogenic efficacy of hCG was evaluated by analysing the expression of vascular markers in OVCAR-3 cells overexpressing hCG. OVCAR-3 cells transfected with the phCMV1-hCGβ vector showed a significant increase in the expression levels of CD31, VEGF, and factor VIII, compared with untransfected and mock-transfected cells. Overexpression of hCG also resulted in a twofold enhancement of HIF-1α expression (Fig. 3).

### Expression of the hCG receptor (hCG-R) in OVCAR-3 cells

**Fig. 4:**
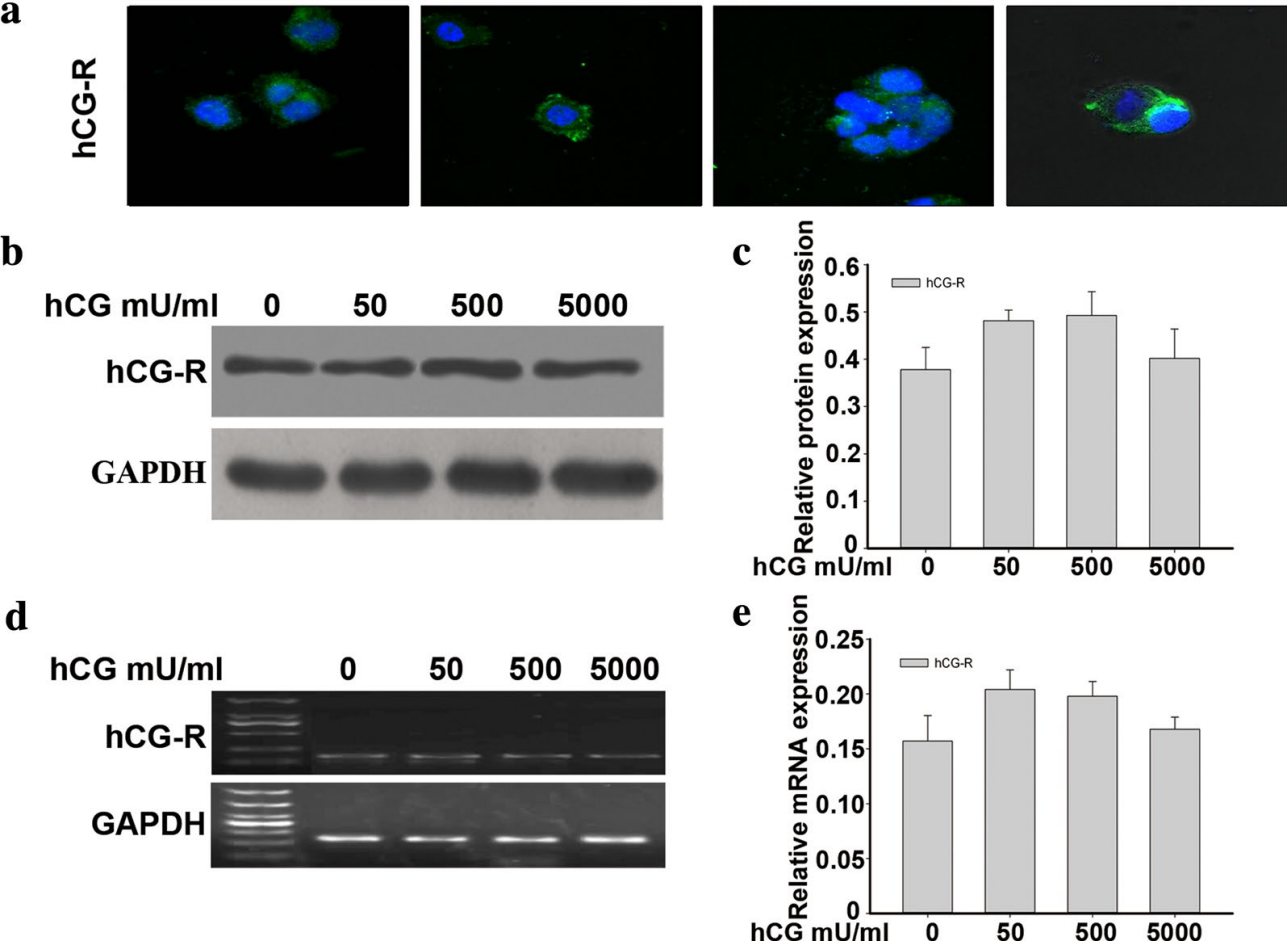
Expression of the hCG receptor (hCG-R) in the ovarian cancer cell line OVACR-3. **a** Confocal image of OVCAR-3 cells following immunofluorescence staining with an hCG-R antibody. Green fluorescence was localized to the periphery of OVCAR-3 cells.* Blue* fluorescence (PI) was used to demonstrate the nucleus. **b**, **d** Different concentrations of hCG did not significantly affect hCG-R expression in OVCAR-3 cells. **b** Protein expression of hCG-R in OVCAR-3 cells treated with hCG (0, 50, 500, or 5000 mU/ml) for 7 days was detected by western blot analysis. **d** Expression of hCG-R mRNA in OVCAR-3 treated with hCG (50, 500, or 5000 mU/ml) was detected by RT-PCR. **c**, **e** Band densities were quantified by densitometric analysis. The protein and mRNA content measured in 3 independent replicates was quantified and the data are presented as the mean ± SD. The data shown were normalized to GAPDH bands and analysed using 1-way ANOVA. p > 0.05

We confirmed that the hCG receptor was expressed in OVCAR-3 cells by confocal microscopy. The green fluorescence was localized to the periphery of OVCAR-3 cells (Fig. 4a). Expression of the hCG receptor in OVCAR-3 cells exposed to 50, 500, or 5000 mU/ml hCG for 7 days were analysed by RT-PCR and western blotting. As shown in Fig. 4b–e, treatment of OVCAR-3 cells with different hCG concentrations did not significantly affect expression of the hCG receptor.

## Discussion

VM is the ability of aggressive cancer cells to acquire an altered phenotype and form a tumour cell-lined vasculature. The tumour cells can express endothelium-associated markers during VM. In tumour vessel channels non-endothelial cells have been found to express typical endothelial markers as seen in uveal melanoma cells which express the endothelial cell markers CD31 and CD34 [23]. Human glioma stem/progenitor cells can transdifferentiate into vascular endothelial cells (VECs) and express VECs markers including CD31, CD34, and vWF significantly under hypoxia [24]. Von Willebrand factor (VWF), a glycoprotein mainly secreted from endothelial cells, is a carrier protein of coagulation factor VIII (FVIII). Factor VIII-associated antigen are vasculogenic mimicry markers [25]. VEGF is a major angiogenesis regulator of human endothelial cells. VEGF appears to contribute to VM formation in some cancer types including ovarian carcinoma [26]. In our study, VM was identified by morphological observations and detection of vascular endothelial cell markers CD31, VEGF, Factor VIII in ovarian cancer cells.

It has been reported that ovarian cancer SKOV3 cells could differentiate into endothelial-like cells and form channels on scaffolds in a microenvironment with low oxygen tension [2]. Our previous findings showed that a microenvironment with hCG localized to scaffolds strongly induced VM in hCG receptor-positive ovarian cancer OVCAR-3 cells, even under normoxic conditions [12].

HCG is a heterodimeric hormone that is primarily produced by the placenta, but is also produced by other normal and cancer tissues at low levels [27, 28]. The human epithelial ovarian cancer cell line OVCAR-3 not only synthesizes hCG, but also expresses hCG receptor on the cell membrane. HCG serves a role in angiogenesis both in vivo and in vitro by increasing capillary formation and endothelial cell migration [29–31]. Berndt et al. [32] demonstrated a direct angiogenic effect of hCG between blastocysts and the maternal endometrium in several experimental models. HCG can also facilitate trophoblast differentiation [33] and positively influence angiogenesis by inducing VEGF and matrix metalloproteinase 9 expression [34]. The angiogenic function of tumour-derived hCG in VM has not been reported.

The angiogenic activity of hCG was investigated here by detecting expression differences in the vascular marker and morphological alterations in OVCAR-3 cells in 3D gels. The addition of exogenous hCG induced expression of vascular markers in OVCAR-3 in a dose-dependent manner, with a dose of 5000 mU/ml hCG in 3D gels showing the strongest influence on vessel-like tube formation by OVCAR-3 cells. These results indicated that hCG potentially affects VM.

In an effort to better understand the involvement of hCG in mediating VM in ovarian cancer cells, siRNA was used to block hCG expression and study its effect on the expression of vascular markers in OVCAR-3 cells. Transfection of the antisense hCG gene resulted in a significant inhibition of vascular cell marker expression in OVCAR-3 cells. OVCAR-3 cells were also transfected with the phCMV1 vector, which drove hCG overexpression and significantly increased vascular cell marker expression. Our data indicated that hCG promotes the trans-differentiation of OVCAR-3 cells into endothelial-like cells.

However, in hCG receptor-negative SKOV3 cells, exogenous hCG failed to induce VM formation. These data suggested that the hormone may act specifically through the hCG receptor. The activity of hCG is initiated by binding of hCG to its transmembrane glycoprotein receptor, which is a member of the G protein-coupled receptor superfamily [35]. Adenylate cyclase on the internal membrane is then stimulated to convert adenosine triphosphate into cyclic adenosine monophosphate [36]. Immunofluorescence staining, RT-PCR, and western blot data detected stable expression of the hCG receptor in the OVCAR-3 cell line. Although OVCAR-3 cells are positive for the hCG receptor, treatment of OVCAR-3 with increasing doses of exogenous hCG had no significant effect on expression of the hCG receptor, suggesting other possible regulatory pathways involved in the effect of hCG [37], which should be a subject of future studies.

We also investigated the effect of hCG on HIF-1α expression. Expression of HIF-1α in OVCAR-3 cells was up-regulated following hCG treatment or transfection with the phCMV1 vector, which drove overexpression of hCG. Conversely, attenuating hCG expression in OVCAR-3 cells via siRNA suppressed HIF-1α expression. HIF-1α is a key transcription factor that mediates responses to oxygen deprivation [38]. Hypoxia, an important feature of the tumour microenvironment, is known to mediate tumour VM through HIF-1α [2, 4]. Driesche et al. [39] demonstrated that HIF-1α expression was induced by hCG in luteinizing granulosa cells under both hypoxic and normoxic conditions. Our present data indicated that hCG is an important regulator of HIF-1α and its downstream target, the vascular marker VEGF [40, 41]. We propose that hCG may exert its angiogenic effect through the HIF-1α-VEGF pathway.

## Conclusions

These results may offer new insights into the possible regulatory role of hCG in VM formation in ovarian cancer. The hCG receptor in OVCAR-3 ovarian cancer cells may potentially serve as a novel target in cancer therapy. Further studies are required to evaluate the signal transduction pathways involved in the activity of hCG in VM of ovarian cancer.

## Methods

### HCG treatment in 3D cultures

The human epithelial ovarian cancer cell lines OVCAR-3 and SKOV3 were purchased from the American Type Culture Collection (Manassas, VA). One hundred and fifty microliters of a co-mixture of Matrigel (Becton–Dickinson, Bedford, MA) and McCoy-5A/RPMI1640 (Gibco, Invitrogen, Carlsbad, CA) was dropped onto glass coverslips in 24-well culture plates and allowed to incubate for 30 min at 37 °C in a humidified 5 % CO_2_ incubator. The medium contained 15 % foetal calf serum and was changed every 48 h. Tumour cells (1 × 10^5^) were seeded onto the gels. Tumour cells were then exposed to different concentrations of recombinant hCG (50, 500, or 5000 mU/ml) for 7 days. HCG was obtained from Sigma (St Louis, MO, USA).

### RT-PCR experiments

Total RNA was isolated from the cultured OVCAR-3 cells using the TRIZOL reagent (Invitrogen, San Diego, CA). First-strand cDNA was synthesized from 2 μg total RNA using oligo-dT primer (T_18_) and reverse transcriptase (Promega, Southampton, UK). Amplification of cDNA was performed in a PerkinElmer Thermal Cycler (GeneAmp PCR Instruments Systems, Roche, Branchburg, NJ). We devised primers with the following sequences: β-hCG: 5′-ACATGGGCATCCAAGGAGC-3′, 5′-GGATTGAGAAGCCTTTATTGTGG-3′ (461 bp); hCG receptor: 5′-TCTATGCCCTATCTGGATTCTAC-3′ and 5′-GGTTCCTACTCACGAGGAGTTTA-3′ (156 bp); CD31: 5′-ACCAAGATAGCCTCAAAGTCG-3′ and 5′-CCTTCACCCTCAGAACCTCAC-3′ (370 bp); VEGF: 5′-TCTGGGCTGTTCTCGCTTCGG-3′ and 5′-AGCAGCAAGGCAAGGCTCCAAT-3′ (414 bp); factor-VIII: 5′-CCCACCGTTACTGACTCGCTAC-3′ and 5′-ATGCTTTCATGCAGGTTTCTCC-3′ (392 bp); HIF-1α: 5′-AAGTGTACCCTAACTAGCCG-3′ and 5′-TCACAAATCAGCACCAAGC-3′ (161 bp); and GAPDH: 5′-CCATTTGCAGTGGCAAAG-3 and 5′-CACCCCATTTGATGTTAGTG-3′ (202 bp). PCR amplification was performed using the following thermocycling conditions: 95 °C for 5 min, followed by 40 cycles of denaturation at 94 °C for 1 min, annealing at 56 °C for 45 s (hCG), 58 °C for 1 min (hCG-R), 50 °C for 45 s (CD31), 60 °C for 45 s (VEGF), 60 °C for 45 s (factor VIII), or 60 °C for 30 s (HIF-1α); and then a final extension step at 72 °C for 10 min. All amplified products were separated in 1 % agarose gels, and the bands were visualized by ethidium bromide staining. In order to semi-quantify the expression level of mRNA, the gels were scanned with standard imaging equipment and the images were analysed with an image analysis software. mRNA contents in the three independent replicates were respectively quantified and presented as mean ± SD.

### Western blot analysis

Mouse monoclonal antibodies against VEGF, factor VIII, β-hCG, hCG receptor, and HIF-1α were obtained from Santa Cruz Biotechnology (Santa Cruz, CA, USA). A rabbit monoclonal antibody against CD31 was obtained from Bioworld (Dublin, OH). Cellular proteins were isolated after rinsing cells with ice-cold phosphate-buffered saline (PBS; pH 7.4) and lysing them on ice with a protein-extraction reagent. The proteins were then separated on an 8 % sodium dodecyl sulphide-Tris polyacrylamide gel. Transfer to a polyvinylidene fluoride membrane was performed at 0.27 mA for 2 h. The membranes were blocked overnight with 1× Tris-buffered saline containing 0.1 % Tween 20 and 5 % skim milk, followed by incubation with primary antibody (1:100) for 1 h and a horseradish peroxidase-conjugated secondary antibody (1:1000) for an additional 1 h at room temperature. Immunocomplexes were visualized by electrochemiluminescence. Protein expression was semi-quantified using a Tiannen imager and analysis system (Shanghai, China). Protein contents in the three independent replicates were respectively quantified and presented as mean ± SD.

### Immunofluorescence staining and confocal microscopy observations

After fixing slides with paraformaldehyde, they were rinsed twice in PBST for 5 min. The slides were then immersed in 3 % hydrogen peroxide for 20 min to quench endogenous peroxidase activity. The specimens were pre-blocked for 30 min in bovine albumin serum. Subsequently, the slides were incubated with a rabbit polyclonal antibody against the hCG receptor (Santa Cruz, CA, USA) at a 1:100 dilution for 1 h at room temperature. After washing 3 times in PBS, the slides were incubated with a fluorescein isothiocyanate-conjugated anti-rabbit immunoglobulin (Santa Cruz, CA, USA) at a 1:100 dilution for 1 h at room temperature. Negative controls were prepared by replacing the primary antibody with Tris-buffered saline. Samples known to be positive for the hCG receptor served as positive controls. A Leica DM IRE2 confocal laser scanning system (oil immersion objectives 63´) with a helium ion/green neon laser (543 nm) was used. Images were collected and processed using Leica confocal software 2.0 and Adobe Photoshop 6.0.

### Small interference RNA (siRNA)

HCG siRNAs were synthesized and ligated into the PGPU6/GFP/Neo vector by Jima Biologic Technology Co. (Shanghai, China). The sequence of pSilencer/β-hCG was 5′-CCCGAGGTATAAAGCCAGGTACA-3′. OVCAR-3 cells were seeded in 6-well plates and grown to 70–90 % confluency in the absence of antibiotics. Transfections were performed with 0.8 μg of the silencing plasmid PGPU6/GFP/Neo-β-hCG and 2 μl Lipofectamine™ 2000 (Invitrogen, Carlsbad, CA, USA), following the manufacturer’s recommended protocol. Control cells were mock-transfected. At 24 h post-transfection, the transfection efficiency was assessed by fluorescence microscopy, revealed that 80 % of the transfectants were positive for green fluorescent protein expression. Stably transfected cells were selected in G418 (0.4 mg/ml; Merck, Darmstadt, Germany) for approximately 2 weeks. The efficiency of β-hCG silencing was analysed by RT-PCR and western blotting.

### Construction of the phCMV1 vector expressing β-hCG (phCMV1-hCGβ)

The recombinant phCMV1-hCGβ plasmid was constructed based on the phCMV1 vector (Gene Therapy System), which encodes the cytomegalovirus mediated-early promoter plus intron A, followed by the SV40 polyA expression cassette. The vector expressing hCG was generated by cloning the sequences encoding hCG into the phCMV1 vector, using unique restriction endonuclease sites. PCR amplification of hCG was performed using the sense primer 5′-CGGAATTCTCCAAGGAGCCGCTTCGG-3′ and the antisense primer 5′-CGGGATCCTTGTGGGAGGATCGG-3′. OVCAR-3 cells were transfected with 2 μg of DNA using 6 μg Lipofectamine™ 2000 (Invitrogen, Carlsbad, CA, USA), according to the manufacture’s guidelines. The resistant clones were selected in G418 (800 μg/ml) for 7 days and expanded in 300 μg/ml G418.

### Statistical analysis

All experiments were performed at least 3 times. The results are presented as the mean ± standard deviation (SD). The data were analysed using SPSS 16.0 for Windows software (SPSS, Inc., Chicago, IL). One-way analysis of variance (ANOVA) was performed to identify statistical differences.

## Abbreviations

3D: 3-dimensional
ANOVA: analysis of variance
hCG: human chorionic gonadotropin
hCG-R: human chorionic gonadotropin receptor
HIF-1α: hypoxia-inducible factor-1α
RT-PCR: reverse transcription-polymerase chain reaction
siRNA: small interfering RNA
VEGF: vascular endothelial growth factor
VM: vasculogenic mimicry
